# *Octosporaconidiophora* (Pyronemataceae) – a new species from South Africa and the first report of anamorph in bryophilous Pezizales

**DOI:** 10.3897/mycokeys.54.34571

**Published:** 2019-06-10

**Authors:** Zuzana Sochorová, Peter Döbbeler, Michal Sochor, Jacques van Rooy

**Affiliations:** 1 Department of Botany, Faculty of Science, Palacký University Olomouc, Šlechtitelů 27, Olomouc, CZ-78371, Czech Republic Crop Research Institute, Centre of the Region Haná for Biotechnological and Agricultural Research Olomouc Czech Republic; 2 Ludwig-Maximilians-Universität München, Systematische Botanik und Mykologie, Menzinger Straße 67, München, D-80638, Germany Palacký University Olomouc Olomouc Czech Republic; 3 Department of Genetic Resources for Vegetables, Medicinal and Special Plants, Crop Research Institute, Centre of the Region Haná for Biotechnological and Agricultural Research, Šlechtitelů 29, Olomouc, CZ-78371, Czech Republic Universität München München Germany; 4 National Herbarium, South African National Biodiversity Institute (SANBI), Private Bag X101, Pretoria 0001, South Africa National Herbarium, South African National Biodiversity Institute Pretoria South Africa; 5 School of Animal, Plant and Environmental Sciences, University of the Witwatersrand, Johannesburg, PO WITS 2050, South Africa University of the Witwa­tersrand Johannesburg South Africa

**Keywords:** Afromontane forests, bryosymbionts, conidia, cryptic biodiversity, muscicolous parasites, *
Sematophyllum
*, *
Trichosteleum
*, South Africa

## Abstract

*Octosporaconidiophora* is described as a new species, based on collections from South Africa. It is characterised by apothecia with a distinct margin, smooth or finely warted ellipsoid ascospores, stiff, thick-walled hyaline hairs, warted mycelial hyphae and growth on pleurocarpous mosses *Trichosteleumperchlorosum* and *Sematophyllumbrachycarpum* (Hypnales) on decaying wood in afromontane forests. It is the first species of bryophilous Pezizales in which an anamorph has been observed; it produces long, claviform, curved, hyaline and transversely septate conidia. Three other cryptic species of *Octospora* were detected using three molecular markers (LSU and SSU nrDNA and EF1α), but these could not be distinguished phenotypically. These are not described formally here and an informal species aggregate *O.conidiophora* agg. is established for them. The new species and finds of *Lamprosporacampylopodis* growing on *Campylopuspyriformis* and *Neottiellaalbocincta* on *Atrichumandrogynum* represent the first records of bryophilous Pezizales in South Africa.

## Introduction

The family Pyronemataceae is not only highly diverse in terms of morphology but also ecologically (Perry et al. 2007, Hansen et al. 2013). It includes six related genera that obligately grow on bryophytes – *Octospora* Hedw., *Lamprospora* De Not., *Neottiella* (Cooke) Sacc., *Octosporopsis* U.Lindem. & M.Vega, *Octosporella* Döbbeler and *Filicupula* Y.J.Yao & Spooner. These ascomycetes, known as bryoparasitic, bryophilous or bryosymbiotic Pezizales, form ca. 0.2–15 mm broad apothecia or perithecia-like apothecia (in *Octosporella*), coloured in shades of orange or red. They infect their hosts by elaborate infection structures consisting of superficial appressoria and intracellular haustoria (Döbbeler 1980). Together with their hosts, they can be found on various substrates like soil, burnt ground, rocks or bark and wood, both in natural and anthropogenic habitats in arctic to tropical regions (e.g. Benkert 1987; Schumacher 1993; Döbbeler 1997; Egertová et al. 2018).

Only rare reports of bryophilous Pezizales from the African continent are known: *Lamprosporamaireana* Seaver, described on the basis of material from Algeria (Seaver 1914); Octosporatetraspora(Fuckel)Korfvar.aegyptiaca J.Moravec from Egypt (Moravec 1972), later revised by D. Benkert as O.leucolomaHedw.var.tetraspora Benkert, as indicated by his revision label; and *O.kilimanjarensis* J.Moravec, described from Tanzania (Moravec 1997) and later reported from Ethiopia together with a probably undescribed *Octospora* species (Lindemann 2013).

From southern Africa, thus far, no finds of these fungi have been reported and no vouchers are deposited in the South African National Collection of Fungi (PREM; Riana Jacobs-Venter pers. comm.). Surprisingly, during three weeks of our field excursions in KwaZulu-Natal and Mpumalanga, eastern South Africa, in February and March 2018, 39 populations of bryophilous Pezizales (*Octospora*, *Lamprospora* and *Neottiella*) were recorded. Only three of them could be assigned to described species, based on morphological characters, host association and DNA sequencing: *Lamprosporacampylopodis* W.D.Buckley growing on *Campylopuspyriformis* (Schultz) Brid. (two collections) and *Neottiellaalbocincta* (Berk. & M.A.Curtis) Sacc. on *Atrichumandrogynum* (Müll. Hal.) A.Jaeger (one collection). The remaining specimens were separated into six morphospecies. One of them, an undescribed *Octospora* species, growing on pleurocarpous mosses from the family Sematophyllaceae (Hypnales), turned out to be very common and remarkable in several aspects after detailed analysis. The aim of this contribution is to provide a description of this species, clarify its phylogenetic relationships and discuss associated taxonomical problems.

## Methods

### Sample collection and observation

Fungi were collected in February and March 2018 in South African Provinces KwaZulu-Natal and Mpumalanga. The description of *Octosporaconidiophora* is based on 11 collections belonging to the most frequent genotype. Observations of apothecial features were made on vital (marked by *) or rehydrated (†) material mostly in tap water, cresyl blue (CRB), lactophenol cotton blue (LPCB) or lactic acid cotton blue (LACB). Absence of amyloidity of asci was confirmed in Lugol´s solution. Infection structures were observed on rehydrated material. Parts of the host plants (leaves and rhizoids) close to an apothecium were separated, pulled apart, treated with LPCB and studied by light microscopy. The preparations were screened at 100× to 200× magnification for the presence of conidia. Infection structures and conidia usually occurred in the same mounts. Illustrations and measurements of hyphae, appressoria and haustoria, as well as conidia, were done in LPCB. The mosses were identified as hosts, based on the presence of appressoria on leaves or rhizoids. The host species were determined using standard techniques for bryophytes (Magill 1981). Collections are deposited in the Mycological department of the National Museum in Prague (PRM) and the herbarium of the Botanische Staatssammlung München (M).

### DNA extraction, PCR amplification and sequencing

DNA was extracted from dried apothecia by the CTAB method as outlined by Doyle and Doyle (1987). Up to three apothecia were homogenised by a pestle and incubated in 300 μl extraction buffer at 65 °C for one hour; the extract was subsequently purified in chloroform-isoamyl alcohol mixture, precipitated by isopropanol and finally dissolved in water and incubated with RNase for 30 min at 37 °C. DNA quality was checked on agarose gel. Molecular sequence data were generated for three loci: the 28S subunit of ribosomal DNA (LSU) was amplified with primers LR0R and LR6 (Vilgalys and Hester 1990), the 18S subunit of rDNA (SSU) with primers NS1 and NS6 (White et al. 1990) and translation elongation factor-1alpha (EF1α) with primers EF1-983F and EF1–1567R (Rehner and Buckley 2005). PCR was performed with Kapa polymerase (Kapa Biosystems, Wilmington, USA) following a standard protocol with 37 cycles and annealing temperature of 54 °C. The PCR products were purified by precipitation with polyethylene glycol (10% PEG 6000 and 1.25 M NaCl in the precipitation mixture) and sequenced from both directions using the same primers by the Sanger method at Macrogen Europe, Amsterdam, The Netherlands.

### Phylogenetic analysis

Newly generated sequences were assembled, edited and aligned in Geneious 7.1.7. (Biomatters, New Zealand) using the MAFFT plugin, manually corrected and deposited in NCBI GenBank under accession numbers MK569288–MK569376. Datasets were compiled from these and previously published sequences (Table 1), aligned, trimmed in order not to contain too many missing data at the ends and concatenated in Geneious 7.1.7. Bayesian Inference for concatenated data was computed in Mrbayes (ver. 3.2.4; Ronquist et al. 2012) with 2×10^7^ generations, sampling every 1000^th^ tree, in two independent runs, each with 4 chains, the first 50% (10^7^) generations being excluded as burn-in. The most suitable substitution model for each locus was determined in Partitionfinder 2.1.1 (Lanfear et al. 2017) using the AIC corrected for small samples (AICc) and a greedy search. Single-locus phylogenies were computed with similar settings, but with 6×10^6^ MCMC generations and the parameter temp. = 0.01.

**Table 1.** Specimens used for the phylogeny inference and their GenBank accession numbers. Newly generated sequences are MK569288–MK569376.

Taxon	Collection code	LSU	SSU	EF1α
*Lamprosporacampylopodis* W.D.Buckley	48633	MF066054	MK569364	MK569289
*Lamprosporadictydiola* Boud.	ldic	MF754056	MK569365	MF754054
LamprosporaminiataDe Not.var.parvispora Benkert	LMSk	MF066065	MK569366	MF754055
*Lamprosporasylvatica* Egertová & Eckstein	UA1	MG947604	MK569367	MK569290
*Neottiellarutilans* (Fr.) Dennis	46853	MK569313	MK569336	MK569288
*Neottiellavivida* (Nyl.) Dennis	NVZla	MF066068	MK569337	MF754051
*Octosporaaffinis* Benkert & L.G.Krieglst.	OAFZla	MF754075	MK569347	MF754045
*Octosporaconidiophora* Sochorová & Döbbeler	ZE11/18	MK569315	MK569348	MK569291
* Octospora conidiophora *	ZE23/18	MK569324	MK569349	MK569294
* Octospora conidiophora *	ZE45/18	MK569316		MK569296
* Octospora conidiophora *	ZE46/18	MK569317	MK569350	MK569298
* Octospora conidiophora *	ZE48/18	MK569321	MK569351	MK569297
* Octospora conidiophora *	ZE57/18	MK569318	MK569352	MK569295
* Octospora conidiophora *	ZE62/18	MK569323	MK569354	MK569299
* Octospora conidiophora *	ZE63/18	MK569319	MK569355	MK569292
* Octospora conidiophora *	ZE71/18	MK569322	MK569356	MK569293
* Octospora conidiophora *	ZE75/18	MK569320	MK569357	MK569300
* Octospora conidiophora *	ZE77/18	MK569331	MK569353	MK569301
*Octosporaconidiophora* agg. – lineage B	ZE37/18	MK569325	MK569358	MK569302
*Octosporaconidiophora* agg. – lineage B	ZE38/18	MK569329	MK569359	MK569303
*Octosporaconidiophora* agg. – lineage B	ZE51/18	MK569327	MK569362	MK569306
*Octosporaconidiophora* agg. – lineage B	ZE52/18	MK569326	MK569360	MK569304
*Octosporaconidiophora* agg. – lineage B	ZE53/18	MK569328	MK569361	MK569307
*Octosporaconidiophora* agg. – lineage B	ZE65/18	MK569330	MK569363	MK569305
*Octosporaconidiophora* agg. – lineage C	ZE44/18	MK569332	MK569373	MK569308
*Octosporaconidiophora* agg. – lineage C	ZE56/18	MK569333	MK569374	MK569309
*Octosporaconidiophora* agg. – lineage D	ZE69/18	MK569334	MK569375	MK569310
*Octosporaerzbergeri* Benkert	ERZ	MF754068	MK569340	MF754042
*Octosporaexcipulata* (Clem.) Benkert	OExc	MF754062	MK569369	MF754047
*Octosporafissidentis* Benkert & Brouwer	Fis	MF754073	MK569341	MF754044
*Octosporahumosa* (Fr.) Dennis	OHZla	MF754074	MK569343	MF754043
*Octosporaithacaensis* (Rehm) K.B.Khare	OLOi	MF754071	MK569346	MF754053
*Octosporakelabitiana* Egertová & Döbbeler	Oct-Jat	MF754065	MK569372	MF754048
* Octospora kelabitiana *	ZE61/16	MF754064	MK569376	MF754049
*Octosporaleucoloma* Hedw.	Oleu	MF066067	MK569370	
*Octosporaorthotrichi* (Cooke & Ellis) K.B.Khare & V.P.Tewari	HR8	MK569314	MK569342	MK569311
*Octosporaphagospora* (Flageolet & Lorton) Dennis & Itzerott	PHG44	MF754072	MK569344	MF754046
*Octosporapseudoampezzana* (Svrček) Caillet & Moyne	OP1	MF754069	MK569339	MF754050
*Octosporawrightii* (Berk. & M.A.Curtis) J.Moravec	WRIG	MF754070	MK569345	
*Octosporellaperforata* (Döbbeler) Döbbeler	PERF	MF754060	MK569368	MF754052
*Octosporopsiserinacea* Egertová & Döbbeler	DUM20/1	MF754057	MK569338	MF754041
*Otidealeporina* (Batsch) Fuckel	KGOL	MK569335	MK569371	MK569312

Divergence times were estimated with Beast 2.5.1 (Bouckaert et al. 2014) using the LSU and SSU data from our sample set (one sample per species or phylogenetic lineage) and six additional species: *Caloscyphafulgens* (Pers.) Boud., *Scutelliniascutellata* (L.) Lambotte, *Cheilymeniastercorea* (Pers.) Boud., *Aleuriaaurantia* (Pers.) Fuckel, *Pyronemadomesticum* (Sowerby) Sacc. and *Sarcoscyphacoccinea* (Gray) Boud. (all sequences obtained from Beimforde et al. 2014; EF1α was not analysed by these authors and, therefore, not included in our molecular dating). Four calibration points were used for the analysis and the divergence times, together with their confidence intervals, were also taken from Beimforde et al. (2014), namely divergence *Cheilymenia*-*Scutellinia*, divergence *Aleuria*-(*Cheilymenia*+*Scutellinia*), split-off of *Sarcoscypha* and split-off of *Caloscypha*. Monophyly was forced for all of the points except the second one due to an unclear position of the *Octospora* clade. Analysis was run under GTR+I+G substitution model (as for Mrbayes), with relaxed clock log normal model and 10^8^ MCMC generations, but the first 50% were excluded as burn-in. Priors included the Yule model with uniform birth rate and exponential gamma shape. Convergence and stationarity were analysed using Tracer v1.7.1 (Rambaut et al. 2018) and results were considered when effective sample size (ESS) ≥ 1000. Statistical uncertainty of divergence time estimates was assessed through the calculation of highest probability density (HPD) values.

## Results

### Phylogenetic and phenotypic analysis

**Figure 1.**
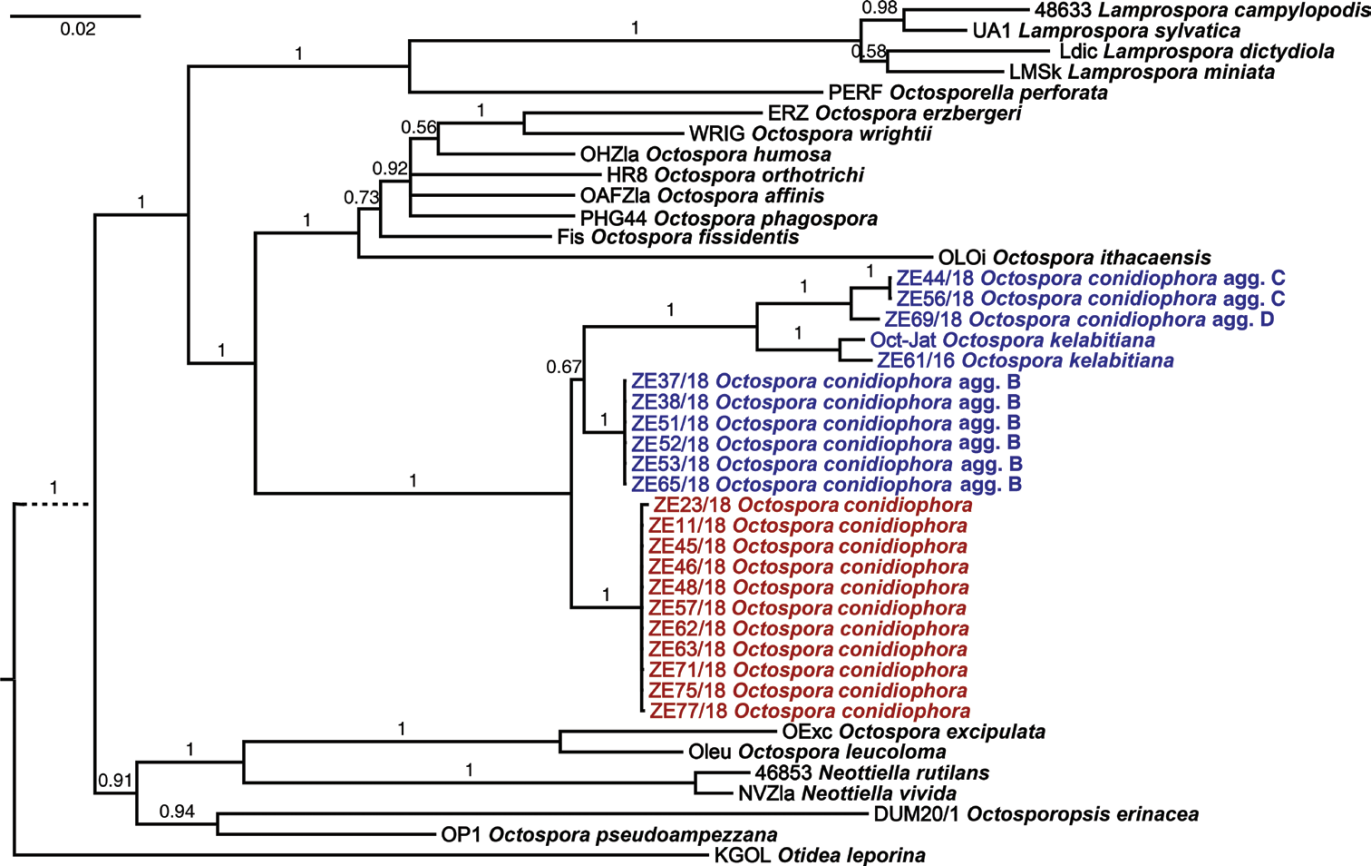
Bayesian phylogeny inference, based on concatenated alignment of EF1α, LSU and SSU sequences. Bayesian posterior probabilities are shown above branches; *Otidealeporina* serves as outgroup; trees based on analysis of each locus are shown in Suppl. material 2: Fig. S1.

**Figure 2.**
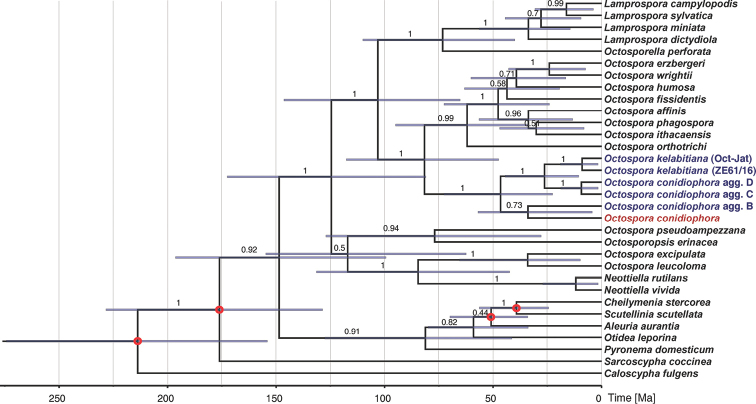
Maximum clade credibility tree with estimated divergence times, based on SSU and LSU data; calibration points are marked by red circles, posterior probabilities shown above branches, bars indicate the 95% highest posterior density (HPD) intervals.

After trimming, the total length of the concatenated alignment was 2702 bp (539 bp from EF1α, 1102 bp from LSU and 1061 bp from SSU, including gaps). Every studied locus provided sufficient polymorphism both amongst and within previously phenotypically delimited groups (Suppl. material 1: Table S1). Four distinct phylogenetic lineages were detected in the concatenated data, as well as in single-locus data within the group of specimens that were hosted by Sematophyllaceae (Fig. 1, Suppl. material 2: Fig. S1). Divergence between them was between 4 and 59 nucleotide differences at every locus (Suppl. material 1: Table S1). The four South African lineages formed a highly supported and distinct clade together with *O.kelabitiana* (Fig. 1). Molecular dating analysis estimated the basal split of bryophilous Pezizales to be 87–172 Ma old (95% confidence interval; mean = 149 Ma), the basal split of the South African accessions was estimated at 23–73 Ma (mean = 47 Ma; Fig. 2).

No significant differences in phenotypic traits were detected amongst the South African lineages using standard characters and methods. They shared the structure of excipulum, stiff, thick-walled hyaline hairs, ellipsoid hyaline ascospores which can be either smooth or ornamented with fine warts and which contain 1 or 2 guttules, warted mycelial hyphae, appressoria, haustoria and presence of anamorph. Although differences amongst individual collections were observed, phenotypic characters did not correspond to the molecular markers and many characters exhibited variability both amongst and within the four phylogenetic lineages (Table 2).

**Table 2.** Variability of selected characters in *O.conidiophora* agg.

Voucher	Ornament of ascospores	(†) Size of ascospores [μm]	(†) Mean size of ascospores [μm]	(†) Q of ascospores	(†) Qm	Observation of conidia	Host
**Lineage A - *Octosporaconidiophora* s. str.**							
ZE11/18	smooth	14.4–16.0 × 8.0–9.1	15.1 × 8.4	1.65–1.91	1.79	yes	* S. brachycarpum *
ZE23/18	smooth	14.0–16.3 × 7.5–9.0	14.9 × 8.0	1.66–2.03	1.84	yes	* T. perchlorosum *
ZE45/18	smooth	13.6–16.2 × 7.5–8.3	15.2 × 7.9	1.74–2.08	1.92	yes	* T. perchlorosum *
ZE46/18	smooth	15.0–17.0 × 8.0–9.9	15.9 × 8.7	1.63–2.06	1.82	yes	* T. perchlorosum *
ZE48/18	smooth	14.5–17.0 × 8.3–9.9	16.1 × 9.0	1.64–1.99	1.79	yes	* T. perchlorosum *
ZE57/18	smooth	14.9–16.2 × 7.9–9.0	15.5 × 8.2	1.69–1.99	1.87	yes	* T. perchlorosum *
ZE62/18	smooth	14.0–16.7 × 8.0–8.9	15.2 × 8.2	1.72–1.99	1.85	yes	* T. perchlorosum *
ZE63/18	smooth	14.0–17.0 × 8.0–9.2	15.4 × 8.7	1.67–1.89	1.77	yes	* T. perchlorosum *
ZE71/18	warted	13.0–15.0 × 8.0–9.9	14.1 × 8.9	1.39–1.73	1.59	no	* T. perchlorosum *
ZE75/18	smooth	14.0–16.1 × 7.8–9.1	15.1 × 8.4	1.65–1.94	1.79	yes	* T. perchlorosum *
ZE77/18	warted	13.5–15.4 × 8.7–10.5	14.4 × 9.4	1.40–1.67	1.53	yes	* T. perchlorosum *
all specimens		13.0–17.0 × 7.5–10.5	15.2 × 8.5	1.39–2.08	1.79		
**Lineage B**							
ZE37/18	warted	13.3–15.5 × 8.3–9.9	14.6 × 9.1	1.47–1.82	1.60	yes	* S. brachycarpum *
ZE38/18	warted	12.5–15.1 × 8.0–9.7	13.8 × 8.8	1.46–1.78	1.57	no	* T. perchlorosum *
ZE51/18	warted	13.5–15.7 × 8.4–10.2	14.5 × 9.4	1.45–1.65	1.54	yes	* T. perchlorosum *
ZE52/18	warted	13.5–16.0 × 8.0–9.5	14.7 × 8.8	1.47–1.83	1.66	yes	* T. perchlorosum *
ZE53/18	warted	14.0–15.3 × 8.0–10.1	14.7 × 9.3	1.47–1.85	1.56	no	* T. perchlorosum *
ZE65/18	warted	13.5–16.2 × 7.7–9.9	14.4 × 8.8	1.47–1.75	1.60	yes	* T. perchlorosum *
all specimens		12.5–16.2 × 7.7–10.2	14.5 × 9.1	1.45–1.85	1.59		
**Lineage C**							
ZE44/18	warted	13.5–15.9 × 7.2–8.1	14.6 × 7.8	1.71–2.01	1.87	yes	* S. brachycarpum *
ZE56/18	warted	13.1–15.4 × 7.0–8.2	14.1 × 7.6	1.66–2.11	1.84	yes	* S. brachycarpum *
both specimens		13.1–15.9 × 7.0–8.2	14.3 × 7.7	1.66–2.11	1.86		
**Lineage D**							
ZE69/18	smooth or lightly warted	13.5–18.0 × 7.9–9.9	15.5 × 8.6	1.61–2.02	1.80	yes	* S. brachycarpum *

### Taxonomy

#### Octospora conidiophora

Taxon classificationFungiPezizalesPyronemataceae

Sochorová & Döbbeler sp. nov.

MB829095

**Figure 3.**
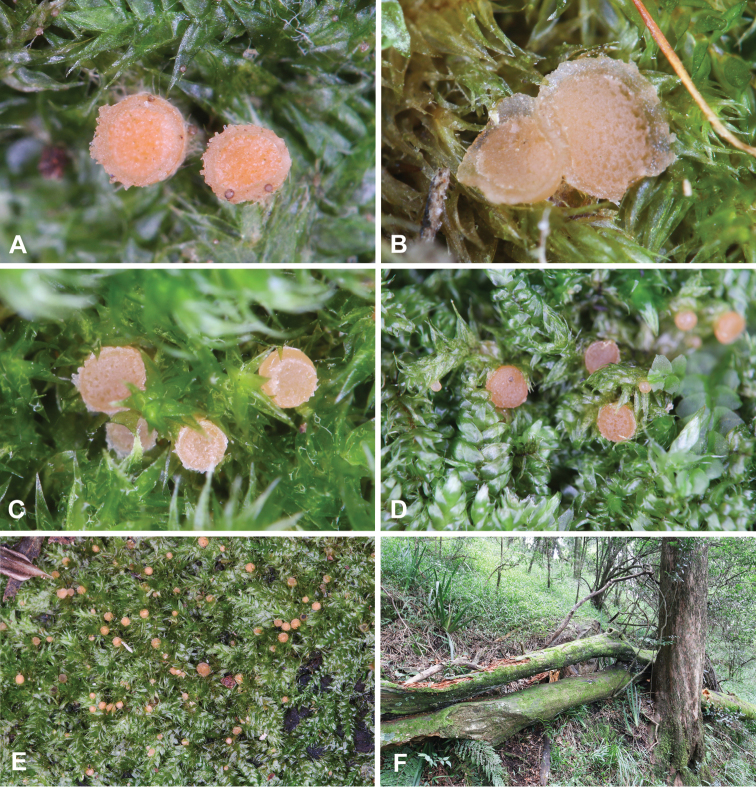
*Octosporaconidiophora*. **A–E** Apothecia in situ **F** Habitat **A, E** ZE63/18 **B** ZE57/18 **C, F** ZE11/18 **D** holotype ZE48/18.

**Figure 4.**
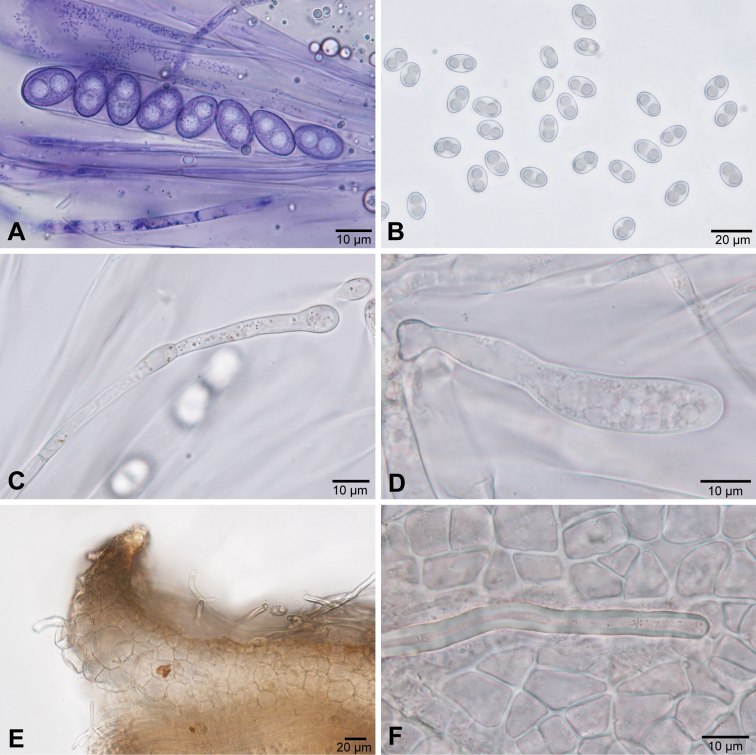
Microscopic characters of *Octosporaconidiophora*. **A** Ascospores inside ascus stained with CRB**B** Free ascospores in tap water **C** Paraphyse in tap water **D** Young ascus in tap water **E** Margin of apothecium in tap water **F** Hair and excipular cells in tap water **A–F** ZE77/18.

**Figure 5.**
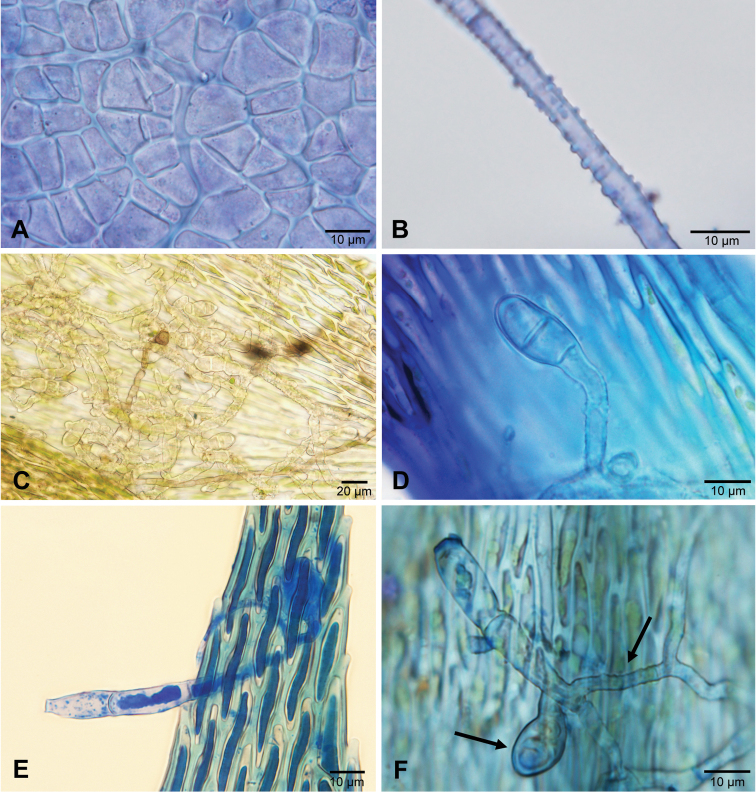
Microscopic characters of *Octosporaconidiophora*. **A** Cells of the outermost layer of excipulum from an outside view stained with CRB**B**Mycelial hypha stained with CRB**C** Appressoria and hyphae on a leaf of *Sematophyllumbrachycarpum* in tap water **D** Appressorium stained with LACB**E** Germinating conidium stained with LPCB**F** Germinated conidium produced a bifurcate warted hypha (right arrow), appressorium (left arrow) probably not connected to the conidium, in LACB**A, B, F** ZE77/18 **C, E** ZE11/18 **D** holotype ZE48/18.

**Figure 6.**
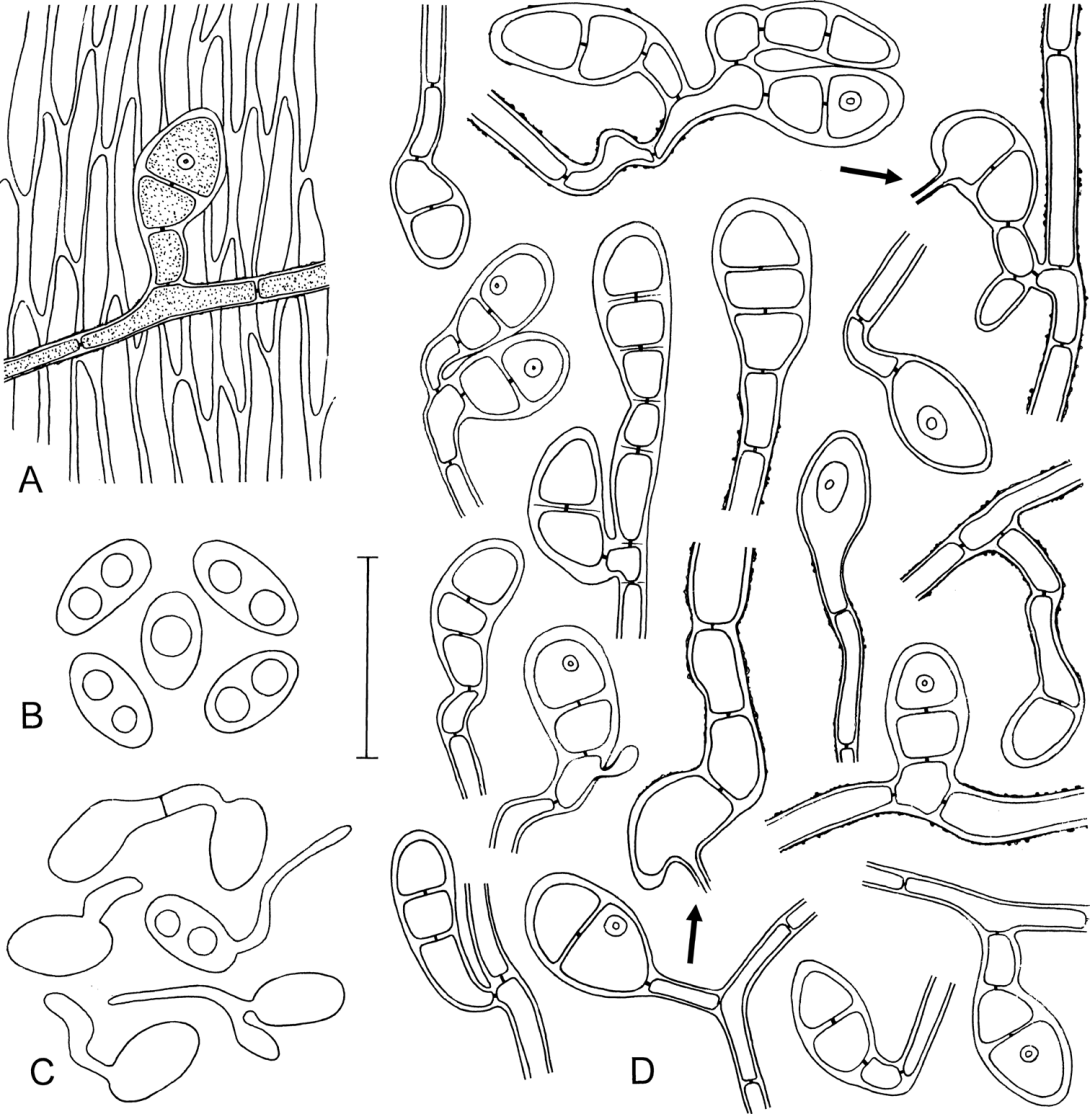
Microscopic characters of *Octosporaconidiophora*. **A** Hypha with two-celled appresorium closely attached to the cells of the host leaf **B** Ascospores **C** Germinating ascospores found on leaves **D** Variation of appressoria mostly seen from above, infection pegs not always observed, appressoria seen in lateral view with infection pegs (indicated by arrows) **A, B, D** holotype ZE48/18 **C** ZE11/18. Scale bar: 30 µm. Illustrated by P.D.

**Figure 7.**
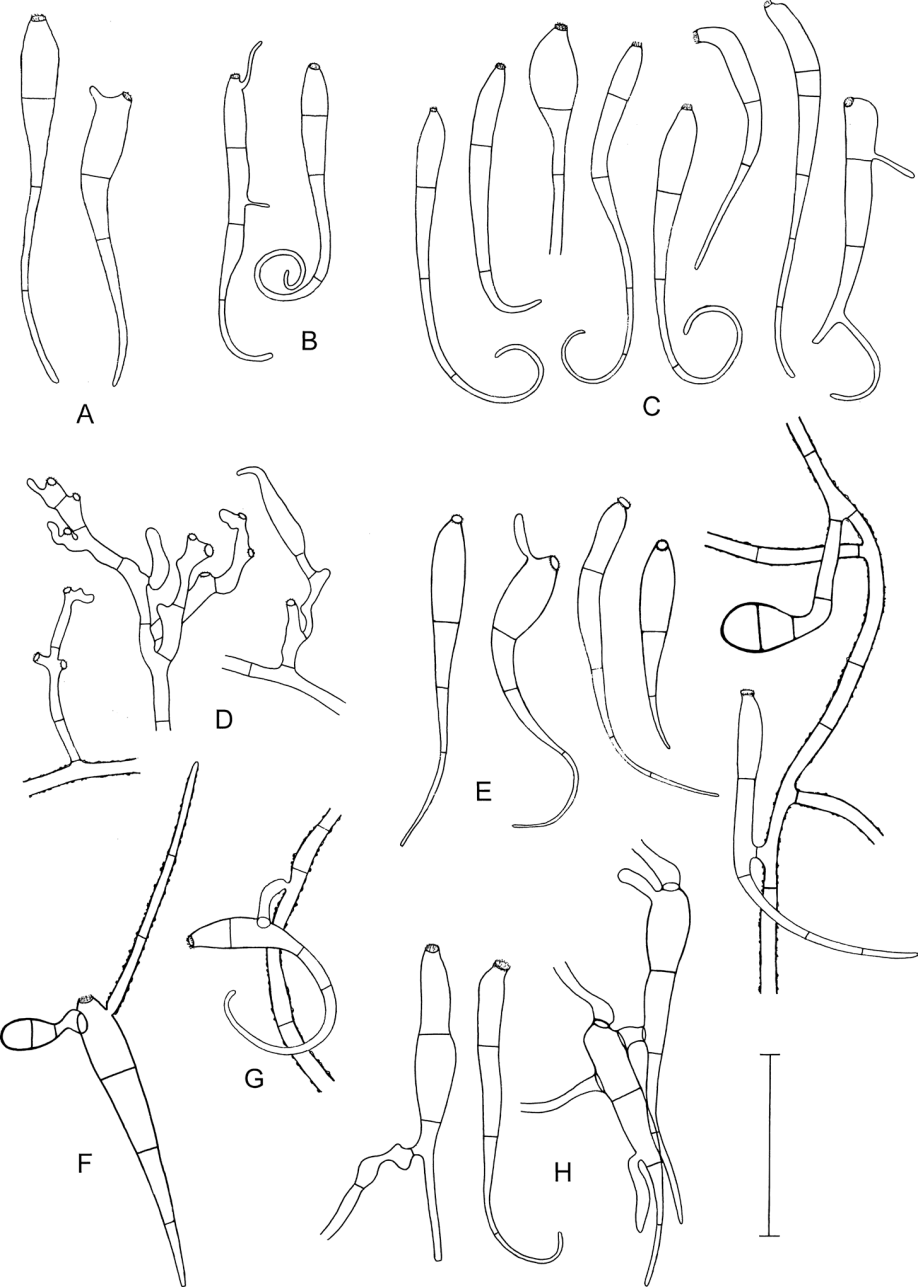
Microscopic characters of *Octosporaconidiophora*. **A–C, E–H** Conidia, distal curved part apparently sometimes broken off, some conidia germinating **D** Conidogenous cells, on the right with a developing conidium **E** (on the right) Conidium anastomosing to mycelial hypha with two-celled appressorium **F** Conidium germinating by a hypha with a warty surface and a two-celled appressorium **G** Conidium with anastomosis to mycelial hypha **H** (on the right) Two germinating conidia with an anastomosis between them **A, F** ZE63/18 **B** ZE46/18 **C** ZE77/18 **D, E** holotype ZE48/18 **G** ZE57/18 **H** ZE11/18. Scale bar: 50 µm. Illustrated by P.D.

**Figure 8.**
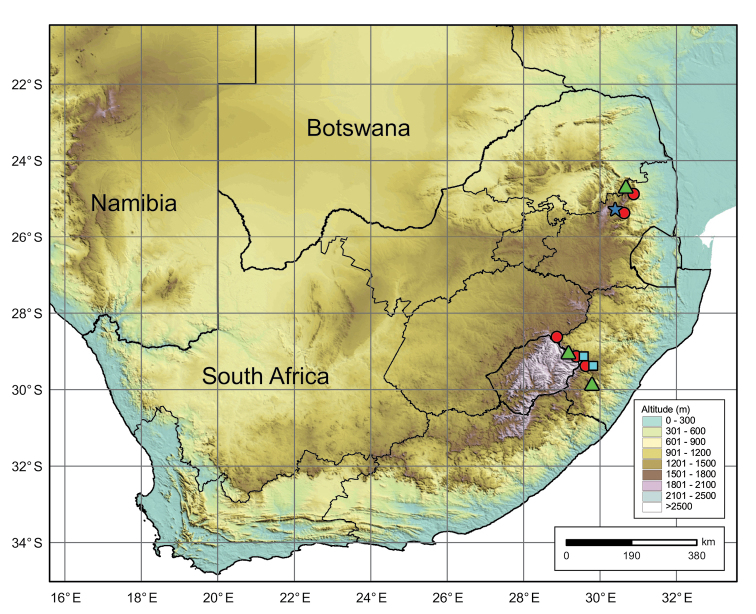
Geographical distribution of the four lineages of *Octosporaconidiophora* agg. in South Africa. Red circle: lineage A (O. con*idiophora* s.str.); green triangle: lineage B; light blue square: lineage C, dark blue star: lineage D.

**Figure 9.**
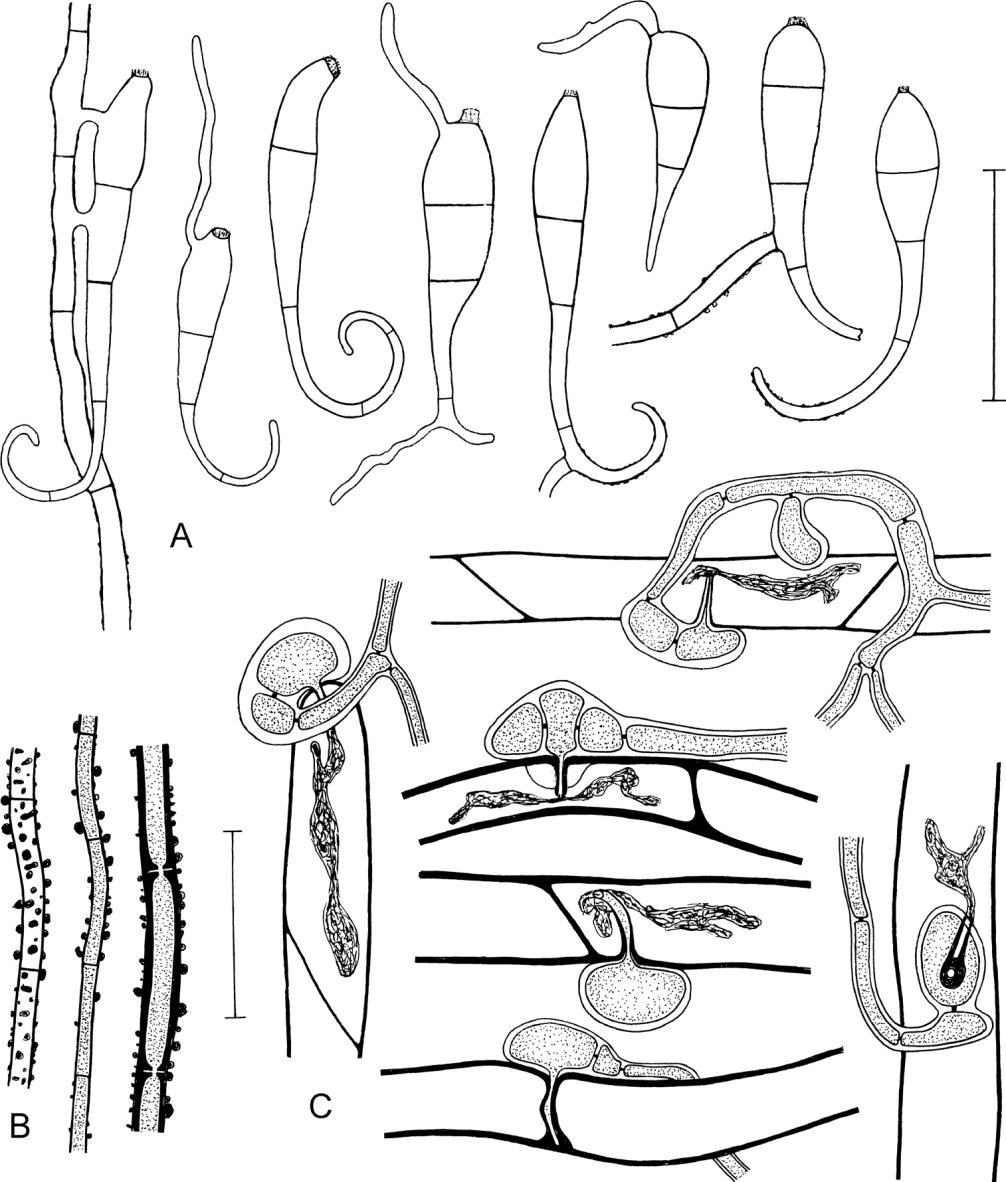
Microscopic characters of *Octosporaconidiophora* agg. (lineage B). **A** Conidia, distal curved part apparently sometimes broken off, five conidia germinating by formation of usually a single hypha, conidium on the left connected to a hypha by two anastomoses **B** Strongly warted hyphae, the left one seen from above, the two others in optical section **C** Appresssoria infecting rhizoids in lateral view, the right one seen from above, infection pegs surrounded by lignituber-like tubes formed by the host cell wall, intracellular haustoria present apart from the lowermost infection where the peg is completely encapsulated by the host cell wall **A, B, C** ZE37/18. Scale bars: 50 µm (**A**); 30 µm (**B, C**). Illustrated by P.D.

Figs 3 , 4 , 5 , 6 , 7 , 8 , 9

##### Etymology.

*Conidiophorus* (Gr./Lat.) refers to production of conidia.

##### Diagnosis.

Differs from *Octosporakelabitiana* by larger apothecia with a distinct margin, infection of pleurocarpous mosses of the family Sematophyllaceae and frequent formation of a *Spermospora*-like anamorph.

TYPE: SOUTH AFRICA. KwaZulu-Natal Province: Uthukela District Municipality, uKhahlamba Drakensberg Park, Injasuti, 29°7.72'S, 29°25.27'E, 1750 m alt., on *Trichosteleumperchlorosum* on decaying wood, 2 Mar. 2018, Z. Egertová (Sochorová) and M. Sochor ZE48/18, holotype: PRM 951743, isotype: M; LSU GenBank accession number: MK569321, SSU GenBank accession number: MK569351, EF1α GenBank accession number: MK569297.

##### Description.

*Apothecialfeatures*: Apothecia in groups on plants of *Trichosteleumperchlorosum* or *Sematophyllumbrachycarpum* or between them, 0.2–1.5 mm broad, up to 0.65 mm high, first subglobose with a small apical opening, later hemispherical, turbinate to disc-shaped, pinkish-orange, sessile, mostly with a well-developed margin, outer surface of excipulum with adpressed to shortly protruding hairs or hyphae.

Hairs *55–205 × 4–10.5 µm, scattered at flanks, hyaline, scarcely septate, obtuse, thick-walled, wall *0.5–3.5 µm thick. Excipulum at the base *230–330 µm thick, laterally about 50 µm thick, composed of angular to subangular (triangular, trapezoid, rectangular), globose, subglobose or irregularly shaped cells, *6–43 × 5–42 µm, outermost cells thick-walled (neighbouring cells divided by up to *6 µm broad wall). Margin *60–280 µm broad, consisting of globose, subglobose, pyriform or trapezoid cells, *10–38 × 7–30 µm.

Subhymenium *40–75 µm wide, consisting of densely packed cylindrical cells *3–7 µm wide mixed with angular or irregularly shaped cells, *4.5–8 × 4.5–6 µm. Paraphyses filiform, straight or bent, unbranched, septate, uppermost one or two cells containing little very pale droplets (*0.5–2 µm in diameter), *2.1–3.5 µm broad (†1.5–2.3 µm), terminal cell *19–83 × 3–7 µm (†18–57 × 3–5.5 µm). Asci *146–197 × 12–15.5 µm (†135–192 × 9.5–12.5 µm), cylindrical, unitunicate, operculate, inamyloid, arising from croziers, with 8 uniseriate ascospores. Ascospores *13–17.2 × 7–10.5 µm, mean 15.2 × 9 µm, Q = 1.34–1.99, Qm = 1.69 (†13–17 × 7.5–10.5 µm, mean 15.2 × 8.5 µm, Q = 1.39–2.08, Qm = 1.79), ellipsoid to narrowly ellipsoid, hyaline, containing one or two lipid guttules (up to *8 µm in diameter if one, *4–5.5 µm if two), smooth or ornamented with cyanophilous, very small, obtuse warts 0.1–0.3 µm broad; germinating with a single germ tube.

*Mycelial* (†): Hyphae restricted to the lowermost plant parts, irregularly growing on and between the leaf bases, stems and especially the rhizoids, hyaline, with ramifications and anastomoses, often thick-walled, (2–)3–6(–7) µm in diameter (excluding ornamentation); hyphal surface with minute to large protuberances, in optical section with numerous minute or larger, semi- or subglobose warts or spines, in surface view, these structures sometimes looking like ridges extended perpendicularly to the hyphal axis; largest warts up to 1.5(–2) µm high; hyphae growing within hyphae present; whole hyphal wall slightly cyanophilous, outermost rough part strongly cyanophilous.

Appressoria variable, frequent (even more than 30 per leaf observed) and easy to detect, closely attached to both leaf sides or to rhizoids, colourless, 1-, 2- or 3-celled, from above elliptical, (14–)16–23(–26) µm long, (8–)11–16 µm wide, laterally seen slightly kidney-shaped, (7–)9–13(–16) µm high, with walls up to 2.5(–4) µm thick; surface rough but not warty, cyanophilous; appressorial cytoplasma strongly cyanophilous; appressoria mostly laterally formed on short stalks; stalks often gradually expanding toward the appressorium; perforation of the host cell wall by means of a delicate peg; peg often surrounded by a brown, straight or curved lignituber-like swelling measuring up to 10(–15) × 2–4(–6) µm; rhizoid wall at the perforation point slightly uplifted towards the appressorium; perforation point not always visible from above.

Haustoria within living leaf cells or rhizoidal cells, at first as a thick short filament, later becoming up to 55 µm long, orientated longitudinally in the rhizoid and developing ramifications (in wider rhizoids), rarely filling out the whole host cell; haustorial cytoplasm strongly cyanophilous.

*Anamorph* (†): Conidia variable in shape and size, claviform, hyaline, transversely septate, ca. (50–)70–115(–154) µm long (including the tail); proximal cell usually distinctly wider than the subproximal cell, rarely cells almost cylindrical, both cells measuring together (30–)35–48(–55) × (6–)7.5–12(–15) µm, subproximal cell continuously attenuating into a tail; tail typically curved to curled, 1- or 2-(3-)celled, (15–)30–60(–100) µm long and (1.5–)2(–2.5) µm in diameter at the distal end; proximal cell of the conidia with a conspicuous, circular, slightly protruding, delicately fringed scar, (3–)4(–4.5) µm in diameter, resulting from detachment from the conidiogenous cell; scar sometimes slightly laterally positioned; walls of conidia cyanophilous; the two proximal cells smooth, the tail sometimes warty (like the hyphae); germ tube one (to three) per conidium, arising from the scar or laterally from different regions of the conidia, including the tail cells.

Conidiogenous cells irregularly shaped, shorter and wider than sterile hyphal cells, rich in cytoplasmic content, usually with 1(–2) scars; shape and size of the scars like those at the conidia, also with a delicately fringed margin.

##### Hosts.

*Trichosteleumperchlorosum*, *Sematophyllumbrachycarpum* (Sematophyllaceae, Hypnales)

##### Distribution.

South Africa, Mpumalanga and KwaZulu-Natal Provinces (Fig. 8).

##### Conservation status.

*Octosporaconidiophora* seems to be a common representative of the genus in South Africa, widespread and forming abundant populations. Its hosts are also common and widespread in the region (see below). Although the main habitat (afromontane forest) is naturally fragmented, it is often protected against human activities by nature reserves or national parks. Therefore, *O.conidiophora* does not fulfil the criteria for categories CR (critically endangered) to NT (near threatened) and we propose its evaluation as LC (least concern) for the present moment.

##### Additional specimens examined.

South Africa. Mpumalanga Province: Ehlanzeni District Municipality, Graskop Gorge, 24°56.74'S, 30°50.8'E, 1355 m alt., on *Trichosteleumperchlorosum* on decaying wood, 6 Mar. 2018, Z. Egertová and M. Sochor ZE62/18 (PRM 951745); Ehlanzeni District Municipality, Graskop Gorge, 24°56.88'S, 30°50.75'E, 1435 m alt., on *Trichosteleumperchlorosum* on decaying wood, 6 Mar. 2018, Z. Egertová and M. Sochor ZE63/18 (PRM 951746); Ehlanzeni District Municipality, Buffelskloof Nature Reserve, 25°15.98'S, 30°31.08'E, 1725 m alt., on *Trichosteleumperchlorosum* on decaying wood, 10 Mar. 2018, Z. Egertová and M. Sochor ZE75/18 (PRM 951748); Ehlanzeni District Municipality, Buffelskloof Nature Reserve, 25°16.37'S, 30°30.62'E, 1605 m alt., on *Trichosteleumperchlorosum* on decaying wood, 9 Mar. 2018, Z. Egertová and M. Sochor ZE71/18 (PRM 951747); Ehlanzeni District Municipality, Buffelskloof Nature Reserve, 25°16.53'S, 30°30.25'E, 1625 m alt., on *Trichosteleumperchlorosum* on decaying wood, 10 Mar. 2018, Z. Egertová and M. Sochor ZE77/18 (PRM 951749). KwaZulu-Natal Province: Uthukela District Municipality, Royal Natal National Park, 28°40.88'S, 28°55.73'E, 1760 m alt., on *Sematophyllumbrachycarpum* on decaying stem, 19 Feb. 2018, Z. Egertová and M. Sochor ZE11/18 (PRM 951739); Uthukela District Municipality, Royal Natal National Park, 28°44.05'S, 28°54.85'E, 1800 m alt., on *Trichosteleumperchlorosum* on decaying stem, 20 Feb. 2018, Z. Egertová and M. Sochor ZE23/18 (PRM 951740). Uthukela District Municipality, uKhahlamba Drakensberg Park, Injasuti, 29°8.95'S, 29°25.35'E, 1665 m alt., on *Trichosteleumperchlorosum* on decaying wood, 3 Mar. 2018, Z. Egertová and M. Sochor ZE57/18 (PRM 951744); Uthukela District Municipality, uKhahlamba Drakensberg Park, Giants Castle Nature Reserve, 29°16.93'S, 29°30.93'E, 1765 m alt., on *Trichosteleumperchlorosum* on decaying wood, 1 Mar. 2018, Z. Egertová and M. Sochor ZE46/18 (PRM 951742); Uthukela District Municipality, uKhahlamba Drakensberg Park, Giants Castle Nature Reserve, 29°16.98'S, 29°30.87'E, 1775 m alt., on *Trichosteleumperchlorosum* on decaying wood, 1 Mar. 2018, Z. Egertová and M. Sochor ZE45/18 (PRM 951741).

##### Data to other lineages.

Lineage B: Mpumalanga Province: Ehlanzeni District Municipality, 3040 m WSW from the Graskop railway station, 24°56.28'S, 30°48.65'E, 1495 m alt., on *Trichosteleumperchlorosum* on decaying wood, 7 Mar. 2018, Z. Egertová and M. Sochor ZE65/18 (PRM 951735). KwaZulu-Natal Province: Uthukela District Municipality, uKhahlamba Drakensberg Park, Injasuti, 29°7.62'S, 29°25.33'E, 1725 m alt., on *Trichosteleumperchlorosum* on decaying wood, 2 Mar. 2018, Z. Egertová and M. Sochor ZE51/18 (PRM 951732); Uthukela District Municipality, uKhahlamba Drakensberg Park, Injasuti, 29°7.57'S, 29°25.38'E, 1715 m alt., on *Trichosteleumperchlorosum* on decaying wood, 2 Mar. 2018, Z. Egertová and M. Sochor ZE52/18 (PRM 951733); Uthukela District Municipality, uKhahlamba Drakensberg Park, Injasuti, 29°7.98'S, 29°26.25'E, 1500 m alt., on *Trichosteleumperchlorosum* on decaying wood, 2 Mar. 2018, Z. Egertová and M. Sochor ZE53/18 (PRM 951734); Sisonke District Municipality, Marutswa Forest, 29°48.55'S, 29°47.28'E, 1465 m alt., on *Sematophyllumbrachycarpum* on decaying stem, 24 Feb. 2018, Z. Egertová and M. Sochor ZE37/18 (PRM 951730); Sisonke District Municipality, Marutswa Forest, 29°48.6'S, 29°47.37'E, 1480 m alt., on *Trichosteleumperchlorosum* on decaying stem, 24 Feb. 2018, Z. Egertová and M. Sochor ZE38/18 (PRM 951731).

Lineage C: KwaZulu-Natal Province: Uthukela District Municipality, uKhahlamba Drakensberg Park, Giants Castle Nature Reserve, 29°17.02'S, 29°30.87'E, 1780 m alt., on *Sematophyllumbrachycarpum* on decaying wood, 28 Feb. 2018, Z. Egertová and M. Sochor ZE44/18 (PRM 951736); Uthukela District Municipality, uKhahlamba Drakensberg Park, Injasuti, 29°8.33'S, 29°25.68'E, 1565 m alt., on *Sematophyllumbrachycarpum* on decaying wood, 3 Mar. 2018, Z. Egertová and M. Sochor ZE56/18 (PRM 951737).

Lineage D: Mpumalanga Province: Ehlanzeni District Municipality, Buffelskloof Nature Reserve, 25°16.93'S, 30°30.45'E, 1470 m alt., on *Sematophyllumbrachycarpum* on decaying wood, 9 Mar. 2018, Z. Egertová and M. Sochor ZE69/18 (PRM 951738).

##### Taxonomic affinities.

The phylogenetically closest and phenotypically most similar species is *Octosporakelabitiana* described from Borneo, which shares most characters with the African species. It also has apothecia with stiff, thick-walled hyaline hairs, ellipsoid, hyaline ascospores of similar size like *O.conidiophora* († in H_2_O (13.5)14.5–17(18) × 7–8(9) μm, in LPCB (12.5)13–16(17) × (6.5)7–8(8.5) μm), filiform, unbranched paraphyses, smooth appressoria of similar size and even the warted mycelial hyphae, which is a character unknown in any other species of bryophilous Pezizales (Egertová et al. 2018). Nevertheless, it can be distinguished easily by growth on a completely different host – thallose liverworts from the genus *Riccardia* Gray. Furthermore, its apothecia are smaller, often taller than wide and lack a distinct margin. Its appressoria are usually one-celled, less often two-celled, while in *O.conidiophora*, two-celled appressoria are very common and even three-celled ones were found. Anamorph has not been detected in *O.kelabitiana*.

## Discussion

According to the available literature and data from the main South African public fungarium (PREM), bryophilous Pezizales are completely unknown from southern Africa, despite the fact that this is a large and species-rich region, which hosts a very diverse bryoflora (Van Rooy and Phephu 2016). Our initial work revealed that this group of fungi is relatively common and probably also very diverse in southern Africa, despite the fact that the work was carried out in extraordinarily dry (and thus unsuitable) summer. Amongst others, four phenotypically similar, yet molecularly distinct lineages were discovered on two host species (lineages A and B on *Trichosteleumperchlorosum* and *Sematophyllumbrachycarpum*, lineages C and D only on *S.brachycarpum*). This research brings novel insights into evolution and systematics of bryophilous ascomycetes and also raises important questions on taxonomic evaluation of these lineages. Therefore, we briefly discuss the taxonomy of cryptic taxa and suggest a suitable taxonomic solution for our collections. As *O.conidiophora* is the first species of bryophilous Pezizales with a detected anamorph, we also discuss this finding. Finally, diagnostic characters and data on distribution of the host mosses are provided as they may help expand the known distribution area of *O.conidiophora* in the future.

### Taxonomic approach

The four lineages could not be distinguished phenotypically on the basis of characters that are normally studied in bryophilous Pezizales, although genetic differentiation was very high at all of the three studied loci (Suppl. material 1: Table S1). Such great genetic distances are usually observed amongst different species or even genera. The observed genetic distances, together with molecular dating, imply that the phenotypically more or less homogeneous morphotype actually represents a group of several cryptic species that have already become reproductively isolated in the Tertiary (Fig. 2). Similar cryptic diversity is probably quite common in fungi, including many genera of Pezizales, e.g. *Genea* Vittad. (Smith et al. 2006, Alvarado et al. 2016), *Geopyxis* (Pers.) Sacc. (Wang et al. 2016), *Helvella* L. (Nguyen et al. 2013, Skrede et al. 2017), *Terfezia* (Tul. & C.Tul.) Tul. & C.Tul. (Ferdman et al. 2009), *Trichophaea* Boud. (Van Vooren 2016) and *Tuber* P.Micheli ex F.H.Wigg. (Bonuso et al. 2010). In bryophilous Pezizales, intraspecific sequence variability was observed, e.g. in *Octosporopsisnicolai* (Maire) U.Lindem., M.Vega & T.Richt. (Lindemann et al. 2014) and *Octosporakelabitiana* (Egertová et al. 2018). Each of the species comprised two genetic lineages that, nevertheless, were relatively weakly diverged and were therefore not treated taxonomically. Besides the significant genetic distances amongst the South African populations, another fact speaks against the possibility that the four lineages could be treated as a single species; the whole clade includes *Octosporakelabitiana* (Fig. 1), a distinct species from Borneo infecting liverwort *Riccardia*. A widely defined species (i.e. including the four lineages but excluding *O.kelabitiana*) would therefore be paraphyletic.

The current approach of many authors to delimitation of species is based primarily or solely on DNA sequence data and sequence-based diagnoses have become almost a common practice in macromycetes (e.g. Buyck et al. 2016, Leacock et al. 2016, Taşkın et al. 2016, Wang et al. 2016, Korhonen et al. 2018). Some authors even aim to base descriptions of new species on environmental sequence data only (e.g. Hibbett et al. 2011). Although molecular phylogenetics is an excellent tool for evaluation of biodiversity, assignment of scientific binominal to molecularly defined species leads to several practical problems, mainly those related to limited accessibility of the methods for many field mycologists. Especially in developing countries, in which even standard optical microscopy can be barely affordable at the leading institutes, determination of species via DNA sequencing is still a matter for the distant future. This methodological obstacle may soon result (or has already resulted in some groups) in the split of traditional phenotype-based taxonomy and molecular taxonomy. Until recently, molecular taxonomy mostly worked with groups, such as molecular operational taxonomic unit (MOTU; Hibbett et al. 2011), phylogenetic species (O’Donnell et al. 2011), virtual taxon (Öpik et al. 2010) etc. and designated an alphanumeric code to them. Nevertheless, many of the molecular taxa are currently given traditional scientific names, often without studying related, validly described species that cannot be sequenced for various reasons. This process, although justified by the aim of cataloguing of global biodiversity, makes the resulting taxonomy impractical or even unusable for field mycologists (and sometimes also for molecular biologists). Another problem with descriptions of species, based on molecular data, is the fact that the borderline between intraspecific and interspecific molecular variation is often unclear (Thines et al. 2018), dependent on many evolutionary factors (e.g. Leliaert et al. 2014) and may become fuzzy after a more intensive and/or extensive sampling is performed, particularly if only one or few molecular markers are used. Nevertheless, this problem also exists with traditional taxonomy (e.g. Flynn and Miller 1990, Paal et al. 1998, Benkert 2001). One solution to the problems mentioned above is an integrative approach. This takes advantage of both multiple characters (morphology, DNA, ecology etc.) and results in robust, phylogeny-based taxonomy that is accessible to various users (e.g. Araújo et al. 2015, Skrede et al. 2017, Haelewaters et al. 2018).

After thorough consideration of the above-mentioned facts, we decided not to formally describe all of the four discovered cryptic species at the present moment. Instead, we prefer to establish two taxa: *O.conidiophora* (s.str.), which refers to the most common phylogenetic lineage A and the informal taxon *O.conidiophora* agg., which applies to all of the four South African cryptic species, but also to the morphologically distinct and host-specific Bornean *O.kelabitiana*. Although the name *O.kelabitiana* is older and should therefore be selected for the aggregate, we believe that the name *O.conidiophora* agg. better suits the pragmatic purposes of this informal taxon. Our approach enables field mycologists to determine their specimens at least on the aggregate level and, at the same time, preserves a monophyletic taxonomical system. Detailed studies may reveal phenotypic differences between the South African lineages of *O.conidiophora* agg., which can then be formally described as species. Until then, we prefer to leave lineages B, C and D without a Latin binominal.

### Anamorph

Conidia have been reported in several genera of Pezizales. The most frequent type of conidia are amerospores which are produced, e.g. in *Caloscypha* Boud. (Paden et al. 1978), *Desmazierella* Lib. (Hughes 1951), *Iodophanus* Korf (Korf 1958, sub *Ascophanus* Boud.), *Pachyphlodes* Zobel (Healy et al. 2015), *Peziza* Fr. (Berthet 1964a, Paden 1967, 1972), *Ruhlandiella* Henn. (Warcup and Talbot 1989, sub *Muciturbo* P.H.B.Talbot), *Thecotheus* Boud. (Conway 1975), *Urnula* Fr. (Davidson 1950), *Cookeina* Kuntze, *Phillipsia* Berk. (Paden 1975), *Pithya* Fuckel (Paden 1972), *Nanoscypha* Denison (Pfister 1973), *Sarcoscypha* (Fr.) Boud. (Harrington 1990), *Geopyxis* (Paden 1972), *Pyropyxis* Egger (Egger 1984, Filippova et al. 2016) and *Trichophaea* (Hennebert 1973). Staurospores can be found in *Miladinalecithina* (Cooke) Svrček (Descals and Webster 1978). The conidia of *O.conidiophora* can be classified as scolecospores or phragmospores and are therefore unique amongst Pezizales with known teleomorph. In their shape, they resemble the conidia of the anamorphic genus *Spermospora* R. Sprague (Ascomycota, Pezizomycotina), a parasite of grasses (Sprague 1948, Seifert et al. 2011).

Detached conidia were regularly found between the rhizoids and leaves in almost all collections of *Octosporaconidiophora* agg. (with the exception of specimens ZE38/18, ZE53/18 and ZE71/18, probably due to limited material). The distal part of the conidia is sometimes short and straight. It is not clear whether this is an artefact caused by breaking off during preparation, although tail fragments have not been found. Germinating conidia are not rare. Longer germination tubes look like normal hyphae with the characteristic warty surface structure (Figs 5F, 7F). Conidia germinating by a two-celled appressorium (Fig. 7F) or connected to a mycelial hypha by an anastomosis (Figs 7E, G, 9A) have been repeatedly observed. Conidiogenous cells (Fig. 7D) are much more difficult to detect than conidia and have only been found in a few collections. The scars formed by detachment of conidia must not be confused with the ends of torn-off hyphae, which inevitably result during preparation. Fully developed conidia still connected to the conidiogeneous cells have not been found. Apparently, mature conidia easily detach from their conidiogenous cells. A developing, still attached conidium was observed once (Fig. 7D).

*Octosporaconidiophora* agg. is the first case amongst bryophilous Pezizales in which an anamorph has been detected. The absence of records of anamorphic states in other species can be caused either by their real rarity or only by their difficulty in detection. The latter can have many reasons. First, bryophilous ascomycetes, in general, stand rather on the periphery of researchers´ interest (see Döbbeler 1997). Second, anamorphs are usually inconspicuous and therefore not easy to encounter. Even if an anamorph is found, it can be difficult to link it with the corresponding teleomorph, because many fungal species commonly occur together. Moreover, anamorphs and teleomorphs are often formed in different environmental conditions (Kendrick 1979) and often at different times. And third, anamorphs are often studied in aseptic cultures and subsequently cultures are used for confirmation of their identity by molecular methods; unfortunately, cultivation of bryophilous Pezizales seems to be problematic (Berthet 1964b) and is not commonly attempted. Although an anamorph has not been confirmed by cultivation methods in *O.conidiophora* agg., the connection of anamorph and teleomorph is based on the evidence discussed above: conidia were repeatedly found amongst the moss plants near the teleomorph; germinating conidia have hyphae with the same ornamentation as observed in the mycelium bearing apothecia; conidiogenous cells occur on the mycelial hyphae; conidia anastomose with mycelial hyphae; the germlings form appressoria.

### Hosts

***Sematophyllumbrachycarpum* (Hampe) Broth.**

Syn: *Hypnumbrachycarpum* Hampe

**Figure 10.**
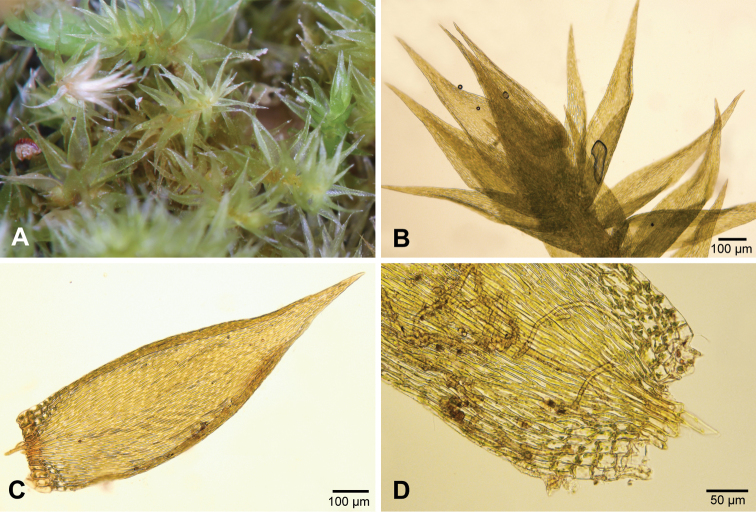
*Sematophyllumbrachycarpum.***A** Plants **B** Typical shoot with leaves **C** Leaf **D** Leaf base with alar cells.

*Sematophyllumbrachycarpum* can be distinguished from other species of *Sematophyllum* in southern Africa by the complanate, straight leaves with relatively large groups of alar cells (in 3–4 rows) that are not much inflated or coloured (Fig. 10, see also Câmara et al. 2019).

**Figure 11.**
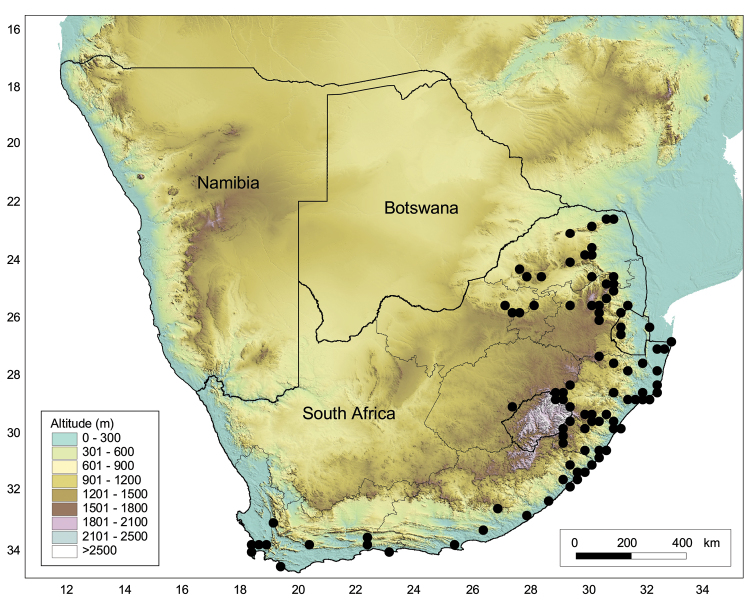
Geographical distribution of *Sematophyllumbrachycarpum* in southern Africa based on records in BM, L, MO and PRE.

The species is by far the most common and widespread species of *Sematophyllum* in South Africa; *S.brachycarpum* is found in forests and wooded areas of the Limpopo, Mpumalanga, North West, Gauteng, Free State, KwaZulu-Natal, Eastern Cape and Western Cape Provinces (Fig. 11, see also Câmara et al. 2019). It occurs as an epiphyte or occasionally on soil or rocks, from sea level up to 1900 m alt. The species is widely distributed throughout the Afromontane Region, as defined by Van Rooy and Van Wyk (2010) and was found to belong to the Widespread Afromontane Subelement, a subdivision of the Afromontane Forest Element (Van Rooy and Van Wyk 2011). The Widespread Afromontane Subelement is centred in the Midlands of KwaZulu-Natal and the Drakensberg escarpment of Mpumalanga as well as in forests in the south-western Cape. The species has also been recorded from Lesotho, Swaziland, Mozambique, Zimbabwe, Zambia, Uganda and Kenya (O’Shea 2006).

***Trichosteleumperchlorosum* Broth. & Bryhn**

**Figure 12.**
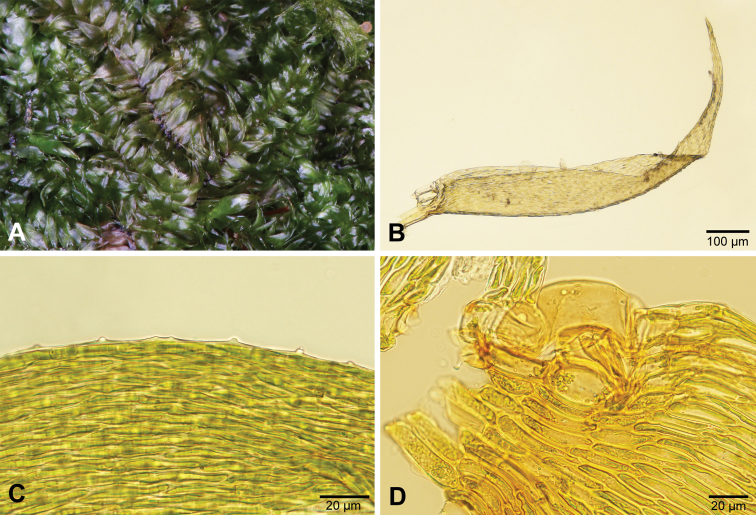
*Trichosteleumperchlorosum.* A. Plants. B. Leaf. C. Leaf papillae. D. Alar cells.

*Trichosteleumperchlorosum* is the only southern African species of Sematophyllaceae (sensu stricto) with papillose leaf cells. However, the papillae are sometimes difficult to see or may be absent on some leaves. The falcate leaves with enlarged, inflated and coloured alar cells will also help to identify the species (Fig. 12, see also Câmara et al. 2019).

**Figure 13.**
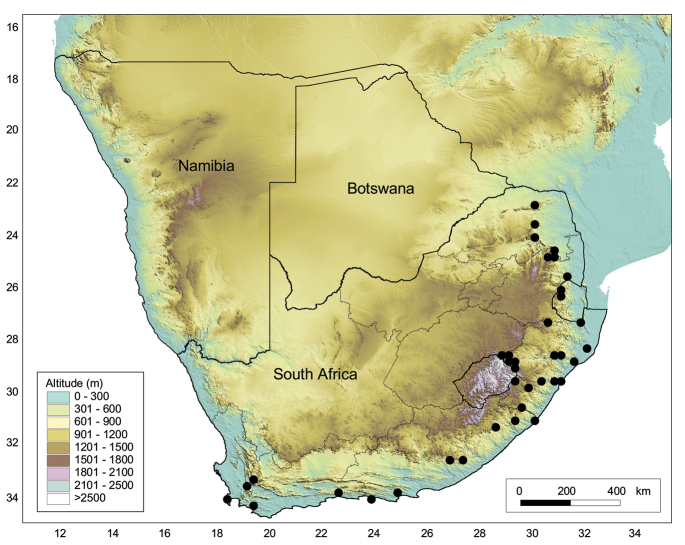
Geographical distribution of *Trichosteleumperchlorosum* in southern Africa based on records in BM, L, MO and PRE.

The species is endemic to the southern part of Africa and occurs as an epiphyte and also on decaying logs or rocks from sea level up to 3090 m high (Drakensberg of KwaZulu-Natal). It is most frequently collected in the KwaZulu-Natal Province of South Africa, but it is also known from Limpopo, Mpumalanga, Eastern Cape and Western Cape Provinces, as well as Swaziland (Fig. 13, see also Câmara et al. 2019). *Trichosteleumperchlorosum* is widespread throughout the Afromontane Region sensu Van Rooy and Van Wyk (2010), but unknown from Afromontane outliers in the Magaliesberg of Gauteng and the North West, the eastern Free State and the Waterberg of Limpopo. It was therefore included in the Tropical Afromontane Subelement (Van Rooy and Van Wyk 2011), which is centred in the Drakensberg escarpment of Mpumalanga and the Midlands of KwaZulu-Natal. This species was also reported from Zimbabwe (O’Shea 2006).

## Supplementary Material

XML Treatment for Octospora conidiophora

## Supplementary materials

Supplementary material 1 Table S1. Distance matrices (nucleotide difference) for each locus of *Octosporaconidiophora* agg. and several randomly selected taxa Data type: molecular data This dataset is made available under the Open Database License (http://opendatacommons.org/licenses/odbl/1.0/). The Open Database License (ODbL) is a license agreement intended to allow users to freely share, modify, and use this Dataset while maintaining this same freedom for others, provided that the original source and author(s) are credited.Zuzana Sochorová, Peter Döbbeler, Michal Sochor, Jacques van Rooy

Supplementary material 2 Figure S1. Bayesian phylogeny inference based on single-locus analyses Data type: phylogenetic tree Explanation note: Bayesian posterior probability are shown above branches. This dataset is made available under the Open Database License (http://opendatacommons.org/licenses/odbl/1.0/). The Open Database License (ODbL) is a license agreement intended to allow users to freely share, modify, and use this Dataset while maintaining this same freedom for others, provided that the original source and author(s) are credited.Zuzana Sochorová, Peter Döbbeler, Michal Sochor, Jacques van Rooy
